# Didactic, practical, good! 20 years of clinical skills training in the German speaking countries

**DOI:** 10.3205/zma001066

**Published:** 2016-08-15

**Authors:** Christoph Stosch, Kai P. Schnabel

**Affiliations:** 1 Universität zu Köln, Medizinische Fakultät, Referat für Lehre, Studium & Studienreform, Kölner Interprofessionelle Skills Labs (KISS), Köln, Deutschland; 2 Universität Bern, Institut für medizinische Lehre, Abteilung für Unterricht und Medien, Bern, Schweiz

## Editorial

In electrical engineering, a transformer controls the level of an AC voltage. “Transformers” are mechanical beings from the planet Cybertron who can continuously transform their body shape. In genetics, transformation describes a process of transferring DNA to another cell, and in medicine, “malignant transformation” means the transition from normal to tumor cell growth. Social transformation refers to the fundamental metamorphosis of a political system into another; in architecture, it means the conversion of existing buildings, and the term “digital transformation” keeps popping up in talk shows. It almost seems as if "transformation" is a ubiquitous phenomenon inherent to the world of things which tags standstill as a failure. The world of medical education is no exception, although if looked at cursorily, the players’ willingness to embrace change may not always and not immediately be noticeable [https://de.wikipedia.org/wiki/Transformation cited 2016 Jan 24]

One particularly successful major project in this area is documented in the present special issue “Clinical skills”: 20 years of development of "skills labs" in the D-A-CH region (Germany/Austria/Switzerland)! Clinical practical skills have been taught before, according to training regulations and laws. However, the fact that an impressive number of laboratories (from Latin laborare = to labor, work, suffer [https://de.wikipedia.org/wiki/Labor cited 2016 Jan 24] have been set up for the systematic and longitudinal training of skills can only be interpreted as part of a transformation process, i. e. the professionalization of medical education.

1996 marked the beginning of the establishment of learning centers for the teaching of patient-centered skills. A hospital rich in history, the Allgemeines Krankenhaus (General Hospital) of Vienna, was first, followed by Berlin (1999), Heidelberg and Frankfurt a.M. (2000), Cologne (2003), Mainz (2003), and others. The accompanying transformation of education in human medicine can be described in many ways: historically (when, what, why), economically (costs, how financed), outcome-oriented (objectives achieved, patients saved), or with regard to the supported processes of change in medical education:

- The transition from pure knowledge-based higher education to a competency-based orientation without systematic inclusion of skills training is simply unthinkable.
- The systematics inherent in action-oriented skills training (“see one – do one”) offers the transition “from teaching to learning” an inherent field for assisted self-regulation in the learning process by providing specific contexts, scenarios and – often formative – feedback.
- The democratization of knowledge through peer teaching often employed in the skills labs finds resonance in the medical faculties by virtue of the changed roles in the learning process.
- The training of practical skills can be seen as practiced, active patient protection.

If form follows function, the transformation of the organization of the skills labs must be - and has been - understood as a response to the professionalization happening in medical education. While it often started as a students‘ initiative, full-time management is today’s standard. In the beginning, the skills part of the curriculum could often only be offered as an added option, but nowadays, curricular integration seems adequate. While at first, skills labs would be housed in buildings set for demolition on the edge of hospital grounds in recognition of the effort spent, new, designated buildings are now being built on prime real estate. 

Formerly, training centers for patient-centered skills developed one-dimensionally toward the fulfillment of tasks in the study of medicine, but today, they are in demand by continuing education and training programs both in medicine and other health professions; hence, they are becoming true nuclei of interprofessional education. 

While formerly, skills lab employees were without outside support, since 2007, they have been able to rely on an established Committee on Practical Skills of the Society for Medical Education which hosts an annual international skills lab symposium (iSLS) of up to 300 participants, and which may be considered as the engine that keeps the described transformation going. Besides providing a platform for scientific exchange, the iSLS focuses on skills lab staff‘s continuing education and networking, with meetings for the student employees, regular skills lab manager meetings (sLiT), and the Committee meetings ultimately serving the professionalization of skills labs in the D-A-CH region. There, the content of the “Consensus Statement on Practical Skills in Medical School” [1] was defined, which had in several respects an unmistakable influence on the development of the National Competence-Based Learning Objectives Catalog in Medicine [http://www.nklm.de cited 2016 April 14]: as a source of ideas for the formulation of “competence levels” and the “milestones of skill acquisition” as well as the basis of chapter 14b, “Clinical practical skills”. Currently, the accreditation regulations appropriate to higher education and concerning education, advanced and continuing training of clinical practical skills are being developed, to be become effective this year.

Finally, this transformation raises the question of further development of centers for learning clinical practical skills. Although the future is open by its very nature, key questions can already be derived whose answers will shape tomorrow‘s discourse:

- Following “competence”, what metaphor will govern the discussion on further development of medical education, and what - deliberate - role will the skills labs play in it?
- What are the effects of the profanation of the skills labs accompanying their professionalization in the context of medical schools (success orientation, competition of means)?
- Furthermore, what is the role of hands-on experience (experience-based learning with models and simulators, and simulated patients) with regard to “augmented reality”, or, in other words: will the digital revolution transform the - as yet physical - skills labs into “holodecks” in the future?

“They say that the ‘little man’ cannot achieve anything. But, if everybody finds another little man, many little men can achieve a lot.” (Thomas Häntsch (*1958), photographer [https://www.aphorismen.de/zitat/108609 cited 2016 July 5].

## Competing interests

The authors declare that they have no competing interests.
